# ﻿Taxonomic status of *Orophea yunnanensis* (Annonaceae), an endemic plant species in Yunnan, China

**DOI:** 10.3897/phytokeys.267.162402

**Published:** 2025-12-02

**Authors:** Bin Yang, Yun-Juan Zuo, You-Bing Wang, Chang-Ji Yang, Yong-Jie Guo, Hong-Bo Ding, Yun-Hong Tan

**Affiliations:** 1 Southeast Asia Biodiversity Research Institute, Chinese Academy of Sciences & Center for Integrative Conservation, Xishuangbanna Tropical Botanical Garden, Chinese Academy of Sciences, Menglun, Mengla, Yunnan 666303, China; 2 Yunnan International Joint Laboratory of Southeast Asia Biodiversity Conservation & Yunnan Key Laboratory for the Conservation of Tropical Rainforests and Asian Elephants, Xishuangbanna Tropical Botanical Garden, Chinese Academy of Sciences, Menglun, Mengla, Yunnan 666303, China; 3 Dali Branch of Yunnan Institute of Forest Inventory and Planning, Dali, Yunnan 671000, China; 4 Baoshan Management Bureau Longyang Branch of Yunnan Gaoligongshan National Nature Reserve, Baoshan, Yunnan 678000, China; 5 Germplasm Bank of Wild Species, Kunming Institute of Botany, Chinese Academy of Sciences, Kunming, Yunnan 650201, China; 6 University of Chinese Academy of Sciences, Beijing 100049, China

**Keywords:** *
Alphonsea
*, Annonaceae, conservation status, molecular phylogeny, new combination, taxonomy

## Abstract

This study elucidates the taxonomic affinity of *Orophea yunnanensis* by considering whether it belongs to *Orophea* or *Alphonsea*. Integrating comparative morphological analysis of type and living materials, field investigations, and molecular phylogenetic evidence, we confirm that *O. yunnanensis* should be transferred to *Alphonsea* as a distinct species. A new combination *Alphonsea yunnanensis* (P.T.Li) Y.H.Tan & Bin Yang is proposed. The mature floral morphology and fruits of *A. yunnanensis* are described for the first time based on wild-collected living specimens and recent herbarium collections. Detailed descriptions, color plates, and geographical distribution, and conservation assessments are provided.

## ﻿Introduction

*Orophea*Blume (1825) and *Alphonsea* Hook.f. & Thomson (1855) belong to tribe Miliuseae in subfamily Malmeoideae (Chatrou et al. 2012, 2018; Nge et al. 2024). *Orophea* comprises about 62 species distributed from the Indian subcontinent through mainland Asia to Southeast Asian islands (Kessler 1988, POWO 2025), with four species recorded in China (Li and Gilbert 2011). It is characterized by the presence of dissimilar petal whorls, its outer petals usually smaller than the inner one, and the inner one being clawed towards the base and generally connivent at anthesis. There is a reduction in number of stamens and carpels per flower and loosely imbricate stamens with a minute connective prolongation not covering the thecae (miliusoid stamens) (Damthongdee et al. 2021, 2024). *Alphonsea* comprises 38 species of shrubs or trees, distributed in wet tropical lowland forests across south and south-east Asia, in India, Sri Lanka, Bangladesh, Myanmar, China, Thailand, Laos, Cambodia, Vietnam, Malaysia, Indonesia, Papua New Guinea and the Philippines (Kessler 1996; Leeratiwong et al. 2020a, 2020b, 2021; Xue et al. 2017; POWO 2025). This genus is easily recognizable by its flower structure, having petals with a saccate base and reflexed apex at anthesis, miliusoid stamens, and multi-seeded monocarps (Kessler 1996; Mols et al. 2004). Seven species have been recorded from China (Li and Gilbert 2011; Xue et al. 2017; Ding et al. 2023; POWO 2025).

**Figure 1.**
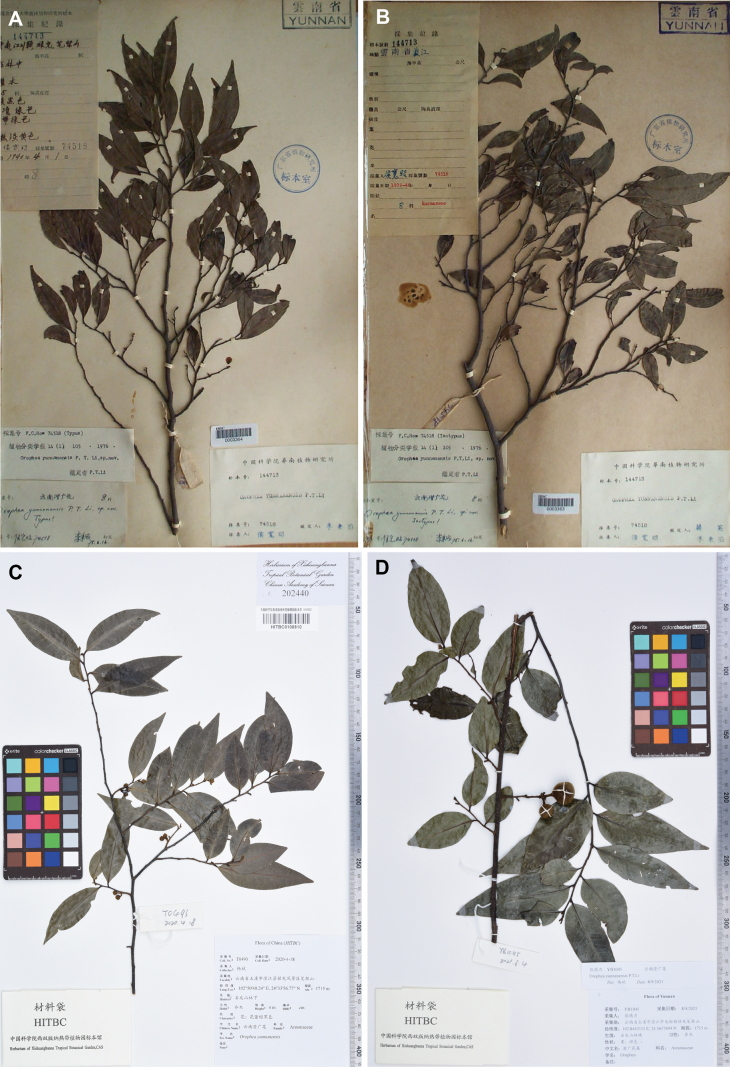
Type specimens and specimens collected from the type locality of *Orophea yunnanensis P.T.Li. **A.** Holotype of *O. yunnanensis (*F. C. How 74518, IBSC [0003364]); **B.** Isotype of *O. yunnanensis (*F. C. How 74518, IBSC [0003363]); **C.** Flowering specimen of *O. yunnanensis (*B.Yang T0493, HITBC[0108810]); **D.** Fruiting specimen of *O. yunnanensis (*D.L. Peng YB1045, HITBC).

*Orophea yunnanensis* P.T.Li (Li 1976) was described based on a bud-stage flowering specimen (*F. C. How 74518*, IBSC) (Fig. 1A, B) collected from Jiangchuan County (now Chengjiang City), central Yunnan Province, southwest China. It remained unrecorded for 76 years after its initial discovery at the type locality in 1940 until its rediscovery in 2016 (Ren et al. 2016). Based on the protologues, the petals of the outer and inner whorls are nearly equal in length, or the inner whorls are slightly shorter, and there are about 12 stamens, the apex of the connective is acute. It is clearly a species with miliusoid stamens. Kessler (1988, 1996) suggested this species might belong to *Alphonsea* rather than *Orophea* during the revision of *Orophea* and *Alphonsea*. On the taxonomy of Annonaceae in China, Hou (2003) tentatively assigned it to *Alphonsea* in his PhD thesis but did not validate the name.

In order to clarify the taxonomic position, we conducted multiple field surveys at the type locality and collected the flowering and fruiting materials of *Orophea yunnanensis* (Fig. 1C, D). After comparing specimens we collected, we found that the leaf blades and flower buds match the type specimens of *O. yunnanensis* very well. Moreover, based on the flowering and fruiting materials, this species should be a member of the genus *Alphonsea*. In order to confirm the generic placement of this species and further elucidate its relationships with other species, a detailed comparison of vegetative morphological characters and phylogenetic analyses was conducted in this study.

## ﻿Material and methods

In this study, measurements and morphological character assessments of *Orophea yunnanensis* and its closely related species were carried out on protologues and previous descriptions (Li 1976; Hou 2003; Li and Gilbert 2011), and our observations on living plants in the field and on herbarium specimens. The general morphological terminology follows Beentje (2016). The specimens deposited in the herbaria A, HITBC, IBK, IBSC, K, KUN, L, NY, P, PE, and US were checked by physical inspection or utilizing high-resolution digital images on the Chinese Virtual Herbarium (https://www.cvh.ac.cn/), GBIF (https://www.gbif.org/) and queried from the respective herbarium websites. The herbarium code is according to the Index Herbariorum (https://sweetgum.nybg.org/science/ih/).

To investigate the phylogenetic position of *Orophea yunnanensis* and the interspecific relationship with closely related species, a total of 22 genera, comprising 88% of the genera within the tribe Miliuseae, along with the genus *Dendrokingstonia* from the tribe Dendrokingstonieae (Chatrou et al. 2012), were included in the phylogenetic analyses based on six plastid regions (*matK*, *ndhF*, *psbA-trnH*, *rbcL*, *trnL-trnF* and *ycf1*). Two *Dendrokingstonia* species were chosen as outgroup, according to comprehensive phylogenetic studies of the family (Chaowasku et al. 2014; Nge et al. 2024). Extensive sampling of *O. yunnanensis* was conducted, with specimens and leaf materials collected from 10 different localities covering most of its distribution range, including the type locality. We also include nine samples representing six *Orophea* taxa and 21 accessions representing ten *Alphonsea* taxa in the phylogenetic analysis to evaluate whether *O. yunnanensis* is genetically affiliated with *Orophea*. Sequences of 28 samples were newly generated in this study (Appendix 2). A total of 267 accessions representing 49 taxa of the tribe Miliuseae were downloaded from GenBank. The voucher information and GenBank accession numbers are provided in Appendix 1.

We utilized ten samples of *Orophea yunnanensis*, 15 samples of *Alphonsea* species and three samples of *Orophea* species for genomic DNA extraction, library construction, and Illumina NovaSeq sequencing through the PersonalBio company (Shanghai, China). The chloroplast genomes were assembled with GetOrganelle v1.7.6.1 (Jin et al. 2020) using default settings and automatically annotated by PGA (Qu et al. 2019) using *Alphonsea hainanensis* (NC_070234.1) as a reference. The resulting sequences of 28 plastomes have been deposited in the GenBase (Bu et al. 2024) in National Genomics Data Center (CNCB-NGDC Members and Partners 2025), Beijing Institute of Genomics, Chinese Academy of Sciences/China National Center for Bioinformation, under accession number C_AA122710.1 to C_AA122737.1 that is publicly accessible at https://ngdc.cncb.ac.cn/genbase. Six plastid regions were extracted from the 29 chloroplast genomes using the script *get_annotated_regions_from_gb.py* (Zhang et al. 2020) and aligned with MAFFT v.7.450 (Katoh and Standley 2013). These regions of all 40 samples were concatenated into one data matrix. The data matrix underlying phylogenetic analysis has been submitted to the Science Data Bank (Yang et al. 2025). Using jModelTest v2.1.10 (Darriba et al. 2012), we assessed the best-fitting model for the concatenated dataset based on the corrected Akaike information criterion (AICc). We conducted the maximum likelihood analysis (ML) under the GTRGAMMA model with RAxML v8.2.12 (Stamatakis 2014). Bayesian inference (BI) was performed with MrBayes v3.2.7 (Ronquist et al. 2012) under the most suitable substitution model selected. Four Markov chain Monte Carlo (MCMC) chains with two independent runs were run for 10^7^ generations, sampling one tree every 1,000 generations. A 50% majority-rule consensus tree was derived after discarding the first 25% trees. The convergence of the two runs was estimated with the average standard deviation of split frequencies < 0.01 and effective sample size (ESS) values > 200 using Tracer 1.6 (Rambaut et al. 2014). Phylogenetic trees were visualized and annotated with FigTree v1.4.3 (Rambaut 2012).

## ﻿Results and discussion

**Figure 2.**
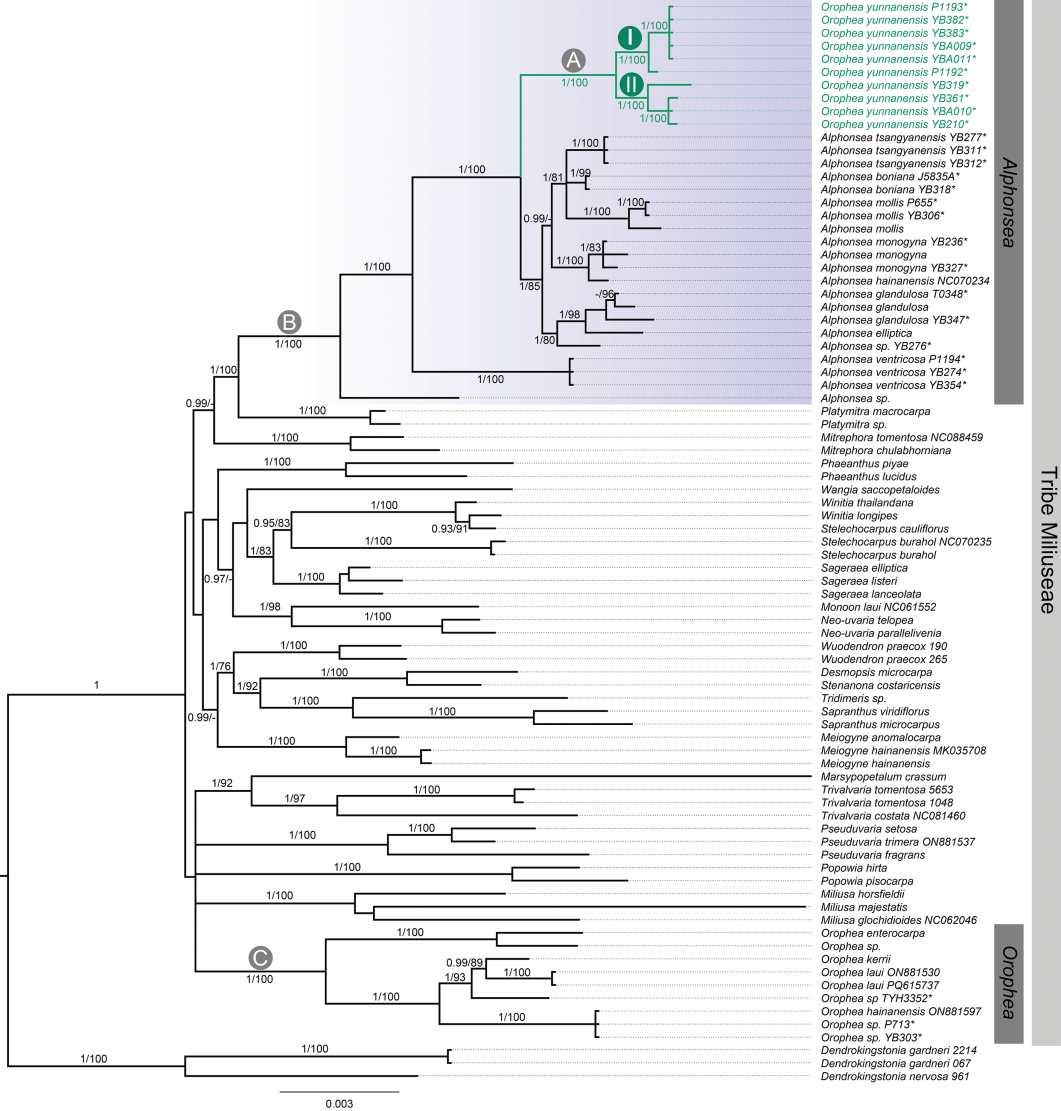
Phylogenetic trees were constructed using maximum likelihood (ML) method and Bayesian Inference based on concatenated dataset of six plastid regions (*matK, *ndhF, *psbA–trnH, *rbcL, *trnL–trnF and *ycf1). Statistical support with bootstrap values (BS) ≥ 70% and posterior probabilities (PP) ≥ 0.95 is presented at each node. Asterisk (*) indicate sequence newly generated in this study.

The aligned matrix for the combined plastid dataset was 6864 bp in length and possessed 727 parsimony-informative sites with gaps treated as missing data. Phylogenetic analysis showed that ten *Alphonsea* species and ten samples of *Orophea yunnanensis* clustered into a strongly supported clade (Fig. 2, Clade B, PP = 1, BS = 100%). This clade was diverged into four major lineages: one *Alphonsea* species from Indonesia, *A. ventricosa*, *O. yunnanensis* (Fig. 2, Clade A), and the remaining eight species of *Alphonsea*. All the samples of *Orophea yunnanensis* form a well-supported clade, as indicated by high statistical values (Fig. 2, Clade A: PP = 1, BS = 100%). Although no significant morphological differences were observed among specimens collected from the ten localities (including sample YBA009 from the type locality, Chengjiang), pronounced genetic divergence was detected within the species, leading to the formation of two distinct subclades: Subclade I (PP = 1, BS =100%) with six samples collected from Chengjiang, Huaning, Jianshui, Mile, Nanjian, Xinping and Subclade II (PP = 1, BS = 100%) with four samples collected from Changning, Fugong, Longyang, Ning’er. Nine samples of the genus *Orophea* formed a monophyletic group with robust statistical support (Fig. 2, Clade C: PP = 1, BS =100%), demonstrating a significant genetic divergence from the genus *Alphonsea*. Molecular evidence derived from plastid regions and morphological evidence have further substantiated the placement of *Orophea yunnanensis* within the genus *Alphonsea*, rather than *Orophea*.

**Figure 3.**
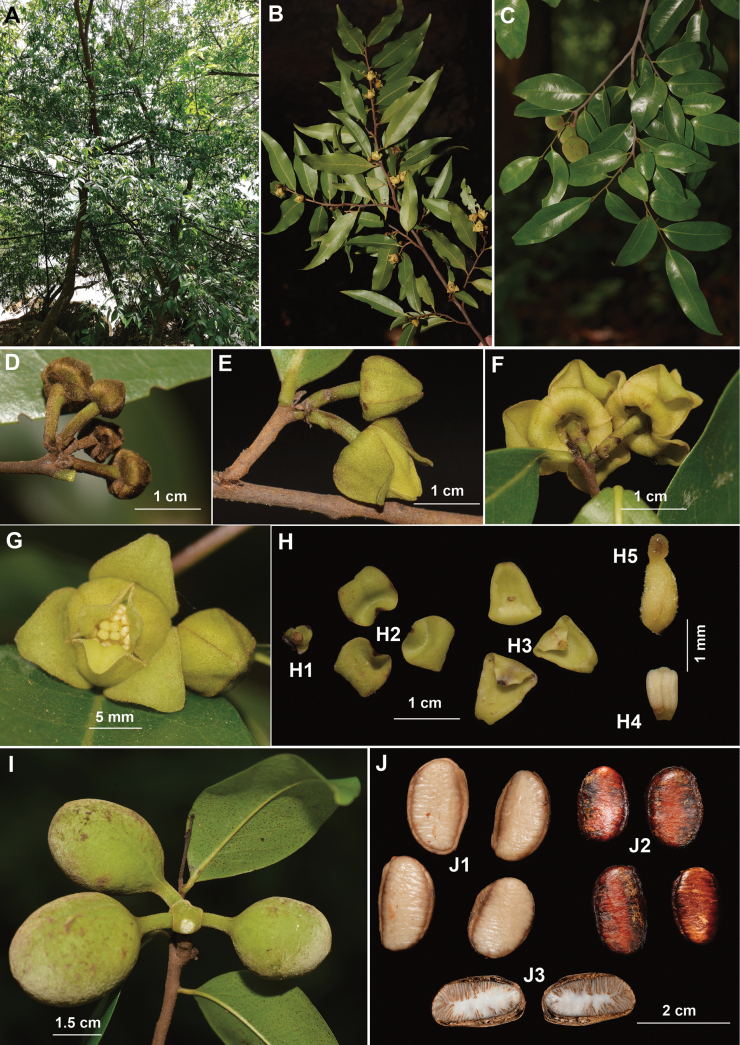
Morphological characters of *Alphonsea yunnanensis. **A.** Habit; **B.** Flowering branch (back view); **C.** Fruiting branch; **D.** Flower buds; **E.** Unopened flowers (side view); **F.** Opened flowers (back view); **G.** Opened flowers (front view); **H.** Dissection of a flower (**H1** sepals **H2** outer petals **H3** inner petals **H4** stamen **H5** carpel); **I.** Fruit; **J.** Seeds (**J1** immature seeds **J2** mature seeds **J3** longitudinal dissection of a seed).

According to our re-examination and comparative analysis of the type specimens, *Orophea yunnanensis* does not belong to *Orophea*, as the typical mitriform inner petals with a narrowly clawed base are absent. Additionally, the petals of both whorls are of the same length, or those of the inner whorl are shorter; this character rarely occurs in *Orophea*, of which the inner petals being usually longer than the outer ones, while conforming well with the known morphology of *Alphonsea*. Moreover, morphological observations and measurements of living plants (Fig. 3) and herbarium specimens revealed that *Orophea yunnanensis* shared a set of morphological characters with *Alphonsea* include petals with a saccate base and reflexed apex at anthesis, miliusoid stamens, and multi-seeded monocarps. Synthesizing previous hypotheses (Kessler 1988, 1996; Hou 2003) and our data, we formally transfer *Orophea yunnanensis* to *Alphonsea* as a new combination. *Alphonsea yunnanensis* resembles *A. tsangyanensis* P.T.Li (Li 1976) in having oblong leaf blade with more than 10 pairs of secondary veins, which likely led to historical misidentifications as *A. tsangyanensis*. However, it can be easily distinguished by smaller flowers, subglobose smooth monocarps with a rounded apex, and larger seeds (Table 1).

**Table 1.** Morphological comparison between *Alphonsea yunnanensis and *A. tsangyanensis. Data on the latter from Li and Gilbert (2011) and our observations.

Characters	*A. yunnanensis	*A. tsangyanensis
Leaf blades	oblong-elliptic to oblong-ovate, 4.2–14.3 × 1.5–4.0 cm	oblong, 6–16 × 2.5–4.5 cm
Number of secondary veins	10–20 pairs	15–19 pairs
Flower size (fresh)	1.0–1.4 cm in diameter	1.5–2.5 cm in diameter
Outer petals (fresh)	9–12 × 6–8 mm	ca. 18 × 10–12 mm
Inner petals (fresh)	11–14 × 5–7 mm	20–22 × 10–11 mm
Carpels	1.5–2 mm long	4–4.5 mm long
Monocarps	subglobose to oblate, apex rounded, smooth	oblong, apex with beak, slightly warty
Seeds	flattened ellipsoid, 1.6–2.1 cm long, 0.9–1.3 cm wide.	flattened ellipsoid to semilunar shape, 1.1–1.5 cm long, 0.7–1.2 cm wide.

### ﻿Taxonomic treatment

#### ﻿ Alphonsea yunnanensis

Taxon classificationPlantaeMagnolialesAnnonaceae

(P.T.Li) Y.H.Tan & Bin Yang comb. nov.

794D85BD-0FA0-57FD-B2B9-D6BCD0B2CA8B

urn:lsid:ipni.org:names:77372726-1

Fig. 3

Orophea yunnanensis P.T.Li, *Acta Phytotax. Sin.* 14(1): 106. 1976. Type: CHINA. Yunnan Province: Jiangchuan County, Luchong, Bijiashan, 1 April 1940, *F. C. How 74518* (holotype: IBSC [0003364], isotype: IBSC [0003363]).
Alphonsea yunnanensis Bân, *Siméon Tén 345* (L [0192066]), nom. nud.
Alphonsea yunnanensis (P.T.Li) X.L.Hou (2003:172), nom. inval. (Art. 30.1).

##### Description.

Small trees, 3–10 m tall, ca. 5–25 cm dbh. Bark grayish black, fissured. Young twigs brownish or grayish black, puberulent to glabrous. Petioles 2–4 mm long, 0.9–1.5 mm in diameter; leaf blades oblong-elliptic to oblong-ovate, 4.2–14.3 × 1.5–4.0 cm, base cuneate to obtuse, apex acute to attenuate, leathery, adaxially sparsely puberulent to glabrous, abaxially glabrous; midrib slightly impressed and sparsely puberulent to glabrous above, raised and sparsely puberulent below; secondary veins 10–20 on each side of the leaf, anastomosing within margin, prominent above, distinctly raised below; tertiary veins reticulate, prominent abaxially and adaxially. Inflorescence leaf-opposed or supra-axillary; 1–4 flowers per inflorescence. Peduncles absent or up to 2.5 mm long. Pedicels (2.0–) 5.5–8 mm long, 0.8–1.0 mm in diameter, puberulous, with one pubescent median or basal bract. Flowers yellow, 1.0–1.4 cm in diameter when fresh. Sepals ovate, 0.7–1.0 (–1.5) × 1.4–1.7(–2.0) mm, pubescent abaxially, sparsely puberulent to glabrous adaxially; outer petals ovate, 9–12 × 6–8 mm when fresh, 7–9 × 5–6 mm when dry, base cuneate to attenuate, apex acute, pubescent abaxially, puberulent adaxially; inner petals narrower, 11–14 × 5–7 mm when fresh, 10–12 × 4.5–5.5 mm when dry, base cuneate, apex acute, puberulous to puberulent abaxially, sparsely puberulent to glabrous adaxially. Stamens “miliusoid” with very short connective prolongation not extending over pollen sacs, 20–30 per flower, ca. 1 mm long, in 3 whorls. Carpels 2–6 per flower, ca. 1.5–2 mm long, puberulous to puberulent; ovules ca. 8 per carpel, biseriate. Fruiting pedicels 6.5–7.8 mm long, 1.7–2.6 mm in diameter; monocarps 1–4 per fruit, subglobose to oblate, 2.1–3.4 cm long, 2.0–2.7 cm in diameter, yellow when mature, shortly pubescent, smooth, apex rounded; stipes 3.5–4.6 mm long, 2–2.6 mm in diameter. Seeds up to 8 per monocarp, flattened ellipsoid, 1.6–2.1 cm long, 0.9–1.3 cm wide, 0.5–0.8 cm thick; endosperm rumination lamelliform.

##### Phenology.

Flowering from January to May, and fruiting from July to September.

##### Distribution and habitat.

**Figure 4.**
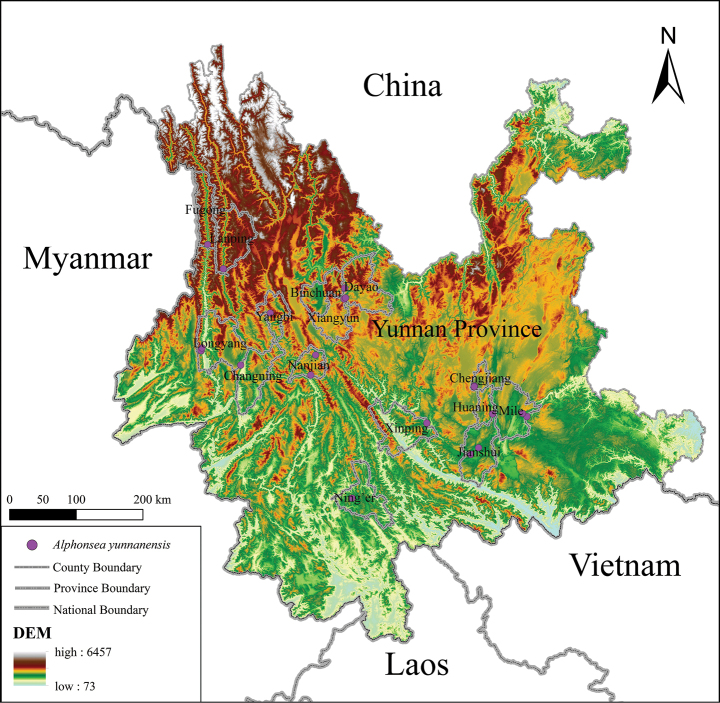
Distribution of *Alphonsea yunnanensis.

According to our field investigation and herbarium records, *Alphonsea yunnanensis* is distributed in several localities of 16 counties and cities across the Yunnan Province (Fig. 4; Chen et al. 2024). It has small trees growing in subtropical limestone evergreen-deciduous broad-leaved mixed forests at 1000–1800 m elevations. As field surveys progress, additional populations are expected to be discovered in its distribution ranges.

##### Conservation status.

This species was previously assessed as critically endangered (CR) (IUCN 2004; Li and Gilbert 2011) and listed as a Plant Species with Extremely Small Populations (PSESP) in Yunnan (Ren et al. 2016; Sun 2021). According to the current distribution of *Alphonsea yunnanensis* (Fig. 4), the extent of occurrence (EOO) for it is 95,142 km^2^ and the area of occupancy (AOO) is 76 km^2^, as calculated by Shiny-GeoCAT (Moat et al. 2023). Based on our initial field investigation, the average number of flowering and fruiting individuals in the wild populations is less than 5. Most distribution points are located on limestone hills or at the edges of river valleys, and they are vulnerable to disturbances. Based on the IUCN Red List Categories and Criteria (IUCN 2024), notably on the number of locations, mature individuals and the AOO, this species qualifies for classification as Endangered (EN, B2ab (iii, v) D).

##### Additional specimens of *Alphonsea yunnanensis* examined.

**China**. Yunnan Province: • **Binchuan City**, Kee y la, via Pe yen tsin ad Pin tchoan (Geyila, 25°55'40.8989"N, 100°56'48.4531"E, 1790 m), 18 April 1917, S*iméon Tén 345* (US[03804827], A[00056744], L[0192066], P[01956285]); • Pay Keau tsui, 13 April 1906, *F. Ducloux 4681* (P[01956282, 01956284]); • 8 February 1909, *F. Ducloux 6752* (L[0192065], P[01956281]); • **Changning County**, ca. 5 km from Xiyi Township to Kejie Town, 7 April 2022, 24°54'13.26"N, 99°23'13.55"E, 1678 m, *Yong-Jie Guo*, *Xiao-Qiang Tao*, *Xing-Xu Sun 22CS22416* (KUN); • **Chengjiang City**, Luchong Village, 24°33'27.50"N, 102°50'21.45"E, 1727 m, 12 March 2014, *Jie Cai*, *Ting Zhang and Chang-Hong Li 14CS9025* (KUN); • Luchong scenic spot, Bijiashan, 24°33'56.77"N, 102°50'40.24"E, 1715 m, 18 April 2020, *Bin Yang T0493* (HITBC); • ibid, 3 May 2020, *Li-E Yang and De-Li Peng T0495* (HITBC); • ibid, 4 August 2021, *De-Li Peng YB1045* (HITBC, PE); • ibid, 24°33'57.58"N, 102°50'39.77"E, 1690 m, 30 August 2023, *Bin Yang and Jian-Guo Chen YB1400* (HITBC); • **Daoyao County**, Sanchahe Town, Rentouguan Village, 25°47'48.30"N, 100°57'25.08"E,1458 m, 17 August 2024, *You-Bing Wang*, *Jian-Jun Yang*, *Hao-Feng Dong YB1793* (HITBC); • **Fugong County**, Pihe Township, 26°32'0.07"N, 98°53'45.52"E,1079 m, 13 March 2024, *Xiao-Qiang Tao YB1573* (HITBC); • **Huaning County**, Panxi Town, Xiaolongtan, 1000 m, 10 August 2016, *En-De Liu*, *Li-Chun Ma and Yun-Hua Xia 4811* (KUN); • ibid, 24°11'4.65"N, 103°5'51.72"E, 1097 m, 12 January 2024, *Bin Yang and Shi-Bo Yuan YB1491* (HITBC); • **Jianshui County**, on the way from Yangjieba to Gantang, 17 March 1941, *Tchen Ngo Liou 18347* (PE[01161779, 01161777], IBSC[0077084]); • Xizhuang Town, Huanglong zen temple, 23°38'55.85"N, 102°45'40.26"E, 1302 m, 30 April 2022, *Bin Yang and Yun-Hong Tan YB1155* (HITBC), ibid, 11 January 2024, *Bin Yang*, *Shi-Bo Yuan YB1489* (HITBC); • **Lanping County**, on the way from Tu’e Township to Zhongpai Township, 1419 m, 11 March 2009, *Shi-Shun Zhou 5254* (HITBC); • **Longyang District**, along the west side of the Lancang Jiang (Mekong River) upstream from Yangbao Bridge on route G320, site of government-closed gold mining operation, 25°28'40"N, 99°20'10"E, 1300 m, 6 August 2019, *David E. Boufford*, *Jian-Ling Guo*, *Lin Su 45549* (P[01105919, 01106103]); • Lujiang Town, Danggang Village, 25°8'11.39"N, 98°48'46.97"E, 1201 m, 27 May 2022, *Ping-Yuan Wang*, *Bin Yang*, *Xiao-Dong Zeng* et al. *T2435* (HITBC); • ibid, 25°5'53.57"N, 98°47'54.44"E, 1119 m, 28 May 2022, *Ping-Yuan Wang*, *Bin Yang*, *Chang-Ji Yang* et al. *T2444* (HITBC); • ibid, Zhanggong Village, Kunmeng river, 25°5'50.48"N, 98°48'0.40"E, 1029 m, 5 April 2023, *Bin Yang*, *Yong-Jing-Wen Yang*, *Jia-Qiang Dong YB1287* (HITBC); • **Mile City**, Xinshao Town, 2 September 2010, *Chong-Jiang Zhao 146* (HITBC[0040077]); • Dongshan Town, 24°10'48.88"N, 103°32'21.19"E, 1357 m, 12 January 2024, *Bin Yang*, *Shi-Bo Yuan YB1492* (HITBC); • **Nanjian County**, Shale Township, Shale Village, Yindian river, 24°45'30.94"N, 100°25'33.33"E, 1273 m, 1 July 2015, *En-De Liu*, *Wei Fang*, *Zhou-Feng Xu* et al. *4402* (KUN); • ibid, 20 March 2020, *Hai-Lei Zhen T0459* (HITBC); • **Ning’er County**, Ning’er Town, Longdong Village, 1500 m, 8 January 2018, *Shi-Shun Zhou 14832* (HITBC); • ibid, 23°5'3.24"N, 101°1'8.87"E, 1432 m, 3 October 2023, *Bin Yang YB1441* (HITBC); • **Xiangyun County**, Midian Town, Chalangshao Village, 25°46'56.22"N, 100°55'58.96"E,1474 m, 17 August 2024, *You-Bing Wang*, *Jian-Jun Yang*, *Hao-Feng Dong YB1792* (HITBC); • **Xinping County**, Yubaiding state forest farm, Yanihe, 24°5'2.82"N, 102°7'39.71"E, 1306 m, 29 March 2020, *Kai-Chun Xiong T0466* (HITBC); • **Yangbi County**, Huimin bridge to Dacun village, 9 November 1946, *Tchen Ngo Liou 22706* (PE[01161778], IBSC[0077088]); • **Yuanjiang County**, exact location unknown, s. d. (presented by Dr. A. Henry, 1901), *A. Henry 13333* (K[003305217], NY[04348761, 04725671]).

## Supplementary Material

XML Treatment for Alphonsea yunnanensis

## ﻿Additional information

### Use of AI

No use of AI was reported.

### Author ORCIDs

Bin Yang https://orcid.org/0000-0001-9791-5159

Yun-Juan Zuo https://orcid.org/0000-0001-9498-6867

You-Bing Wang https://orcid.org/0009-0001-3168-4965

Yong-Jie Guo https://orcid.org/0000-0003-1752-4495

Hong-Bo Ding https://orcid.org/0000-0003-1843-8425

Yun-Hong Tan https://orcid.org/0000-0001-6238-2743

### Data availability

All the data that support the findings of this study are available in the main text, and supplementary materials are available from Journal website.

## ﻿Appendix 1

The list of voucher information and GenBank accession numbers for samples used in this study. Accession numbers for DNA sequences published in previous studies are given in the following format: *matK*, *ndhF*, *trnH-psbA*, *rbcL*, *trnL-trnF*, *ycf1* and chloroplast genome. (– represents missing data).

***Alphonsea elliptica*** Hook.f. & Thomson, AY518807, JQ690401, JQ690402, AY318966, AY319078, JQ690403; ***Alphonsea glandulosa*** Y.H.Tan & B.Xue, KY054909, KY054912, KY054915, KY054906, KY054918, KY054921; ***Alphonsea hainanensis*** Merr. & Chun, –, –, –, –, –, –, NC070234; ***Alphonsea mollis*** Dunn, KY054907, KY054910, KY054913, KY054904, KY054916, KY054919; ***Alphonsea monogyna*** Merr. & Chun, KY054908, KY054911, KY054914, KY054905, KY054917, KY054920; ***Alphonsea* sp.**, MT040200, MT040210, MT040222, MT040233, MT040245, MT040256; ***Dendrokingstonia gardneri*** Chaowasku, MH585797, MH585823, MH585803, MH585807, MH585812, MH585816; ***Dendrokingstonia gardneri*** Chaowasku, KJ418391, KJ418385, KJ418399, KJ418381, KJ418406, KJ418378; ***Dendrokingstonia nervosa*** (Hook.f. & Thomson) Rauschert, MH585798, KJ418386, KJ418400, KJ418382, KJ418407, MH585817;

***Desmopsis microcarpa*** R.E.Fr., AY518804, JX544771, AY841461, AY319059, AY319173, JX544758; ***Marsypopetalum crassum*** (R.Parker) B.Xue & R.M.K.Saunders, HQ286571, JQ723792, KF709057, HQ286577, HQ286583, JQ723929; ***Meiogyne anomalocarpa*** D.M.Johnson & Chalermglin, ON920473, ON920479, –, ON920493, ON920467, ON920507; ***Meiogyne hainanensis*** (Merr.) Bân, –, –, –, –, –, –, MK035708; ***Meiogyne hainanensis*** (Merr.) Bân, MW024839, MW024841, –, MW024847, MW024850, MW024853; ***Miliusa glochidioides*** Hand.-Mazz., –, –, –, –, –, –, NC062046; ***Miliusa horsfieldii*** (Benn.) Baill. ex Pierre, OL438785, OL438811, OL438833, OL438839, OL438861, OL438884; ***Miliusa majestatis*** Damth., Sinbumr. & Chaowasku, OR267124, OR267125, OR267122, OR267126, OR267123, OR267127; ***Mitrephora chulabhorniana*** Damth., Aongyong & Chaowasku, MT040201, MT040211, MT040223, MT040234, MT040246, MT040257; ***Mitrephora tomentosa*** Hook.f. & Thomson, –, –, –, –, –, –, NC088459; ***Monoon laui*** (Merr.) B.Xue & R.M.K.Saunders, –, –, –, –, –, –, NC 061552; ***Neo-uvaria parallelivenia*** (Boerl.) H.Okada & K.Ueda, AY518794, KC857570, KC857571, AY319000, AY319113, KC857572; ***Neo-uvaria telopea*** Chaowasku, JX544751, JX544778, JX544791, JX544755, JX544783, JX544766; ***Orophea enterocarpa*** Maingay ex Hook.f., AY518815, JQ690416, JQ690417, AY319006, AY319119, JQ690418; ***Orophea hainanensis*** Merr., –, –, –, –, –, –, ON881597; ***Orophea kerrii*** Kessler, AY518818, JQ690419, JQ690420, AY319008, AY319121, JQ690421; ***Orophea laui*** Leonardía & Kessler, –, –, –, –, –, –, ON881530; ***Orophea laui*** Leonardía & Kessler, –, –, –, –, –, –, PQ615737; ***Orophea* sp.**, –, JQ723808, –, JQ723869, JQ723922, JQ723948; ***Phaeanthus lucidus*** Oliv., OK664963, OK664961, OK664965, OK664967, OK664969, OK664971; ***Phaeanthus piyae*** Wiya, Aongyong & Chaowasku, OK664962, OK664960, OK664964, OK664966, OK664968, OK664970; ***Platymitra macrocarpa*** Boerl., AY518812, JQ723809, JQ690423, AY319013, AY319127, JQ723949; ***Platymitra* sp.**, JQ690426, JQ690427, JQ690428, –, JQ690425, JQ690429; ***Popowia hirta*** Miq., AY518860, JX544830, JX544806, AY319042, AY319156, JX544816; ***Popowia pisocarpa*** (Blume) Endl. ex Walp., LC737003, JQ723812, LC737576, LC736570, AY319158, JQ723953; ***Pseuduvaria fragrans*** Y.C.F.Su, Chaowasku & R.M.K.Saunders, MW054646, JQ723813, MW054650, MW054654, MW054642, JQ723954; ***Pseuduvaria setosa*** (King) J.Sinclair, KC857582, KC857583, KC857584, EU522334, EU522224, KC857585; ***Pseuduvaria trimera*** (Craib) Y.C.F.Su & R.M.K.Saunders ON881537, –, –, –, –, –, –, ON881537; ***Sageraea elliptica*** (A.DC.) Hook.f. & Thomson, KC857587, OR760025, OR760026, OR760027, OR760028, KC857590; ***Sageraea lanceolata*** Miq., AY518799, JX544774, JX544787, AY319050, AY319164, JX544762; ***Sageraea listeri*** King, OR759991, OR759997, OR760003, OR760009, OR760015, OR760021; ***Sapranthus microcarpus*** (Donn.Sm.) R.E.Fr., AY518806, –, KU727346, AY319052, AY319166, MG874915; ***Sapranthus viridiflorus*** G.E.Schatz, AY743493, AY841422, AY841515, –, AY319165, JQ723955; ***Stelechocarpus burahol*** (Blume) Hook.f. & Thomson, MT040202, MT040213, MT040225, MT040237, MT040248, MT040258; ***Stelechocarpus burahol*** (Blume) Hook.f. & Thomson, –, –, –, –, –, –, NC070235; ***Stelechocarpus cauliflorus*** (Scheff.) R.E.Fr., MT040205, MT040216, MT040228, MT040240, MT040251, MT040261; ***Stenanona costaricensis*** R.E.Fr., AY518801, JX544772, AY841516, AY319069, AY319183, JX544759; ***Tridimeris* sp.**, JX544750, JX544773, JX544786, JX544753, JX544782, JX544761; ***Trivalvaria costata*** (Hook.f. & Thomson) I.M.Turner, –, –, –, –, –, –, NC081460; ***Trivalvaria tomentosa*** B.Xue, Y.H.Tan & Y.S.Chen, MZ066637, MZ066639, MZ066641, MZ066643, MZ066645, MZ066647; ***Trivalvaria tomentosa*** B.Xue, Y.H.Tan & Y.S.Chen, MZ066638, MZ066640, MZ066642, MZ066644, MZ066646, MZ066648; ***Wangia saccopetaloides*** (W.T.Wang) X.Guo & R.M.K.Saunders, MT040204, MT040215, MT040227, MT040239, MT040250, MT040260; ***Winitia longipes*** (Craib) Chaowasku & Aongyong, MT040207, MT040218, MT040230, MT040242, MT040253, MT040263; ***Winitia thailandana*** Chaowasku & Aongyong, MT040208, MT040219, MT040231, MT040243, MT040254, MT040264; ***Wuodendron praecox*** (Hook.f. & Thomson) B.Xue, Y.H.Tan & X.L.Hou, MF687367, MF687369, MF687371, MF687373, MF687375, MF687377; ***Wuodendron praecox*** (Hook.f. & Thomson) B.Xue, Y.H.Tan & X.L.Hou, MF687368, MF687370, MF687372, MF687374, MF687376, MF687378.

## Appendix 2

Voucher information for newly generated DNA sequences is provided using the following format: species, voucher data, and GenBase accession numbers for chloroplast genomes.

****Alphonsea boniana*** Finet & Gagnep., J5835A (**C. Liu* et al.* *13CS6315* [KUN]), C_AA122710.1;

****Alphonsea boniana*** Finet & Gagnep., YB318 (**B.Yang YB1438* [HITBC]), C_AA122727.1;

****Alphonsea glandulosa*** Y.H.Tan & B.Xue, T0348 (**B.Yang T0348* [HITBC]), C_AA122716.1;

****Alphonsea glandulosa*** Y.H.Tan & B.Xue, YB347 (**L. Bai YB1465* [HITBC]), C_AA122730.1;

****Alphonsea mollis*** Dunn, P655(Cultivated at XTBG), C_AA122714.1;

****Alphonsea mollis*** Dunn, YB281 (**D.L.Quan* et al. **L1240* [HITBC]), C_AA122723.1;

****Alphonsea monogyna*** Merr. & Chun, YB236 (Cultivated at SCBG), C_AA122719.1;

****Alphonsea monogyna*** Merr. & Chun, YB327, (Cultivated at SCBG), C_AA122729.1;

****Alphonsea* sp.**, YB276 (**Y.H.Tan* et al. **ML181* [HITBC]), C_AA122721.1;

****Alphonsea tsangyanensis*** P.T.Li, YB277 (**S.S.Zhou* et al. **ZSS19784* [HITBC]), C_AA122722.1;

****Alphonsea tsangyanensis*** P.T.Li, YB311 (**H.B.Ding D196* [HITBC]), C_AA122725.1;

****Alphonsea tsangyanensis*** P.T.Li, YB312 (**H.B.Ding D203* [HITBC]), C_AA122726.1;

****Alphonsea ventricosa*** (Roxb.) Hook.f. & Thomson, P1194 (**B.Yang XTBG0086* [HITBC]), C_AA122713.1;

****Alphonsea ventricosa*** (Roxb.) Hook.f. & Thomson, YB274 (**B.Yang* et al. **WPY1022* [HITBC]), C_AA122720.1;

****Alphonsea ventricosa*** (Roxb.) Hook.f. & Thomson, YB354 (**B.Yang YB1138* [HITBC]), C_AA122731.1;

****Orophea hainanensis***, P713 (Cultivated at XTBG), C_AA122715.1;

****Orophea hainanensis***, YB303 (**Y.H.Tan* et al. **L0777* [HITBC]), C_AA122724.1;

****Orophea* sp.**, TYH3352 (**Y.L.Li & Y.H.Tan TYH3352* [HITBC]), C_AA122717.1;

****Orophea yunnanensis*** P.T.Li, P1192 (**H.L.Zhen T0459* [HITBC]), C_AA122711.1;

****Orophea yunnanensis*** P.T.Li, P1193 (**K.C.Xiong T0466* [HITBC]), C_AA122712.1;

****Orophea yunnanensis*** P.T.Li, YB210 (**B.Yang* et al. **YB1287* [HITBC]), C_AA122718.1;

****Orophea yunnanensis*** P.T.Li, YB319 (**B.Yang YB1441*[HITBC]), C_AA122728.1;

****Orophea yunnanensis*** P.T.Li, YB361 (**X.Q.Tao YB1573* [HITBC]), C_AA122732.1;

****Orophea yunnanensis*** P.T.Li, YB382 (**B.Yang & S.B.Yuan YB1492* [HITBC]), C_AA122733.1;

****Orophea yunnanensis*** P.T.Li, YB383 (**B.Yang & S.B.Yuan YB1491* [HITBC]), C_AA122734.1;

****Orophea yunnanensis*** P.T.Li, YBA009 (**B.Yang YB1045* [HITBC]), C_AA122735.1;

****Orophea yunnanensis*** P.T.Li, YBA010 (**Y.J.Guo* et al. **22CS22416* [KUN]), C_AA122736.1;

****Orophea yunnanensis*** P.T.Li, YBA011 (**B.Yang & Y.H.Tan YB1155* [HITBC]), C_AA122737.1.
